# Possibilities for revascularization and closure of wound defects in lower limbs with critical ischemia through free gracilis flap transfer: review of 3 cases

**DOI:** 10.1590/1677-5449.202500182

**Published:** 2025-10-17

**Authors:** Ilya Igorevich Kalitko, Mikhail Viktorovich Chernyaev, Aleksander Georgievich Faybushevich, Igor Mikhailovich Kalitko

**Affiliations:** 1 “Clinic of Innovation Surgery”, Moscow, Russia.; 2 Peoples’ Friendship University of Russia – RUDN University, Moscow, Russia.

**Keywords:** limb salvage, gracilis flap, free flap, critical ischemia, case report, salvamento de membro, retalho de grácil, retalho livre, isquemia crítica, relato de caso

## Abstract

This article presents three clinical cases of lower limb revascularization with closure of trophic defects using free gracilis flap transfer; two patients with Buerger’s disease and one patient with type 2 diabetes mellitus. In these cases, we also describe and discuss direct or indirect evidence of indirect revascularization of ischemic tissues by means of the flap, as well as the advantages and limitations of the free gracilis muscle flap.

## Resumo

Este artigo apresenta três casos clínicos de revascularização dos membros inferiores com fechamento de defeitos tróficos utilizando transferência livre de retalho do músculo grácil: dois pacientes com doença de Buerger e um paciente com diabetes melito tipo 2. Além disso, são descritas e discutidas as evidências diretas e indiretas da revascularização indireta de tecidos isquêmicos por meio de retalho, bem como as vantagens e limitações do retalho livre do músculo grácil.

## INTRODUCTION

Treatment of patients with critical lower limb ischemia who have trophic lesions constitutes a complex challenge, further complicated by the presence of severe diseases, such as diabetes mellitus or Buerger’s disease, which are often associated with damage to or absence of the distal arterial bed. Consequently, isolated revascularization may not be feasible due to impaired outflow. In such cases, indirect or combined revascularization with bypass decompression can be performed through free flap transfer.^1^

## CASE 1

A 31-year-old male smoker suffering from Buerger’s disease was admitted with complaints of pain in the left foot at rest and wet necrosis of the toes and forefoot.

Notable analyses included total Ig E: 1756 IU/ml; Erythrocyte sedimentation rate: 35 mm/hour; Leukocytes: 9 x 10^9/l; and C-reactive protein: 55.76 mg/l. The wound culture identified multiresistant pseudomonas aeruginosa and alcallogenes faecalis and enterococcus faecalis, which were both sensitive to amoxiclav.

Computed tomography (CT) analysis revealed a superficial femoral artery (SFA) aneurysm, a patent popliteal artery, occlusion of all arteries of the lower leg from the upper third, with corkscrew-shaped collaterals, and collateral filling of the posterior tibial artery (PTA) in the lower third of the leg.

A single-stage operation was performed. Initially, the SFA aneurysm was prosthetically repaired with a PTFE prosthesis, then an autovenous shunt was formed from the popliteal artery to the PTA, using a reversed great saphenous vein with two end-to-end anastomoses, followed by balloon angioplasty of the medial plantar artery. However, high bypass resistance was observed on the control ultrasound, indicating impaired outflow.

A gracilis muscle was taken from the same leg and, after transmetatarsal resection of the left foot and sanitization with antiseptics, it was fixed to the wound.

**Figure 1:**
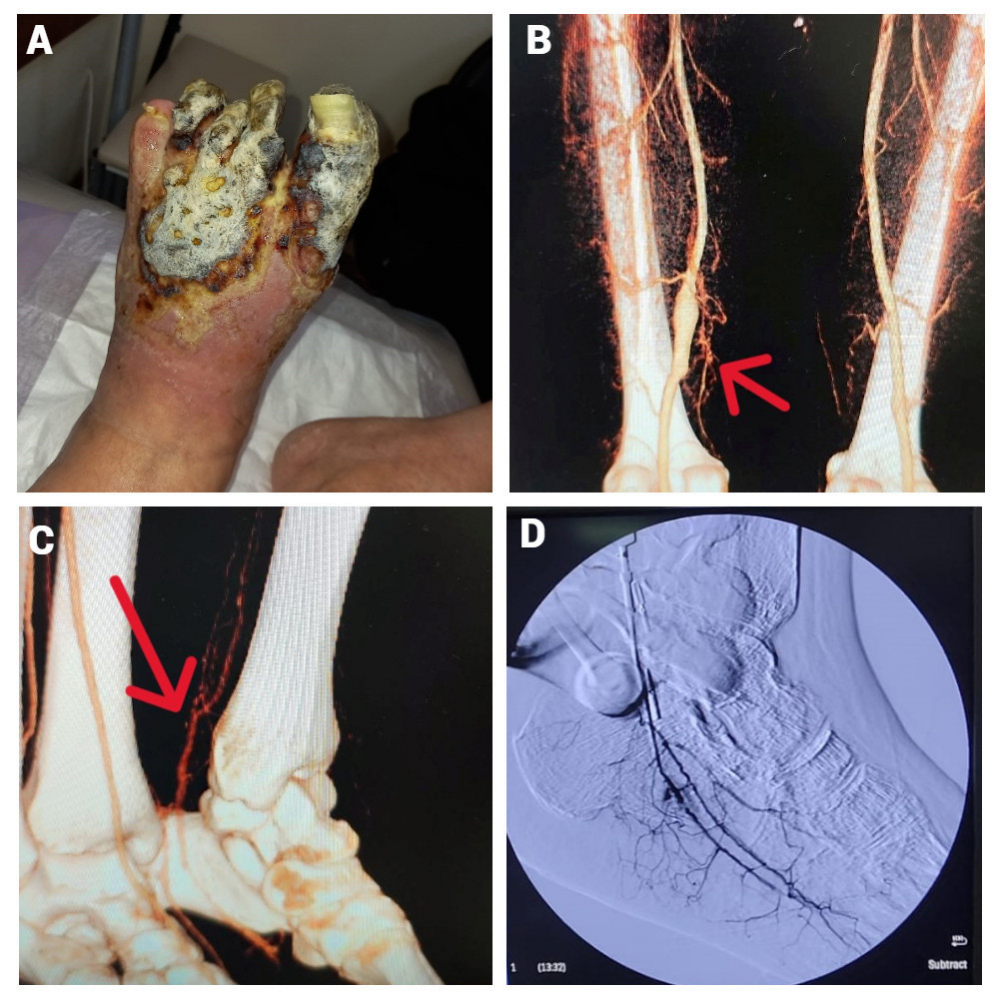
**(a)** Wet gangrene of the left forefoot; **(b)** SFA aneurysm; **(c)** collateral filling of the left PTA in the lower third of the left leg; **(d)** intraoperative angiography of the flap via the autovenous branch. SFA: Superficial femoral artery; PTA: Posterior tibial artery.

Subsequently, microanastomoses were sequentially formed, first end-to-end between the flap vein and the posterior tibial vein and then the flap artery was anastomosed end-to-side to the autovenous bypass, 1.5 cm proximal to the site of the distal anastomosis, between the bypass and the posterior tibial artery (Figure 1).

Postoperatively, the wound was bandaged with chlorhexidine and iodopyron, and ultrasound and thermographic monitoring of flap viability were conducted. One week later, the muscle flap was covered with a split skin graft.

**Figure 2:**
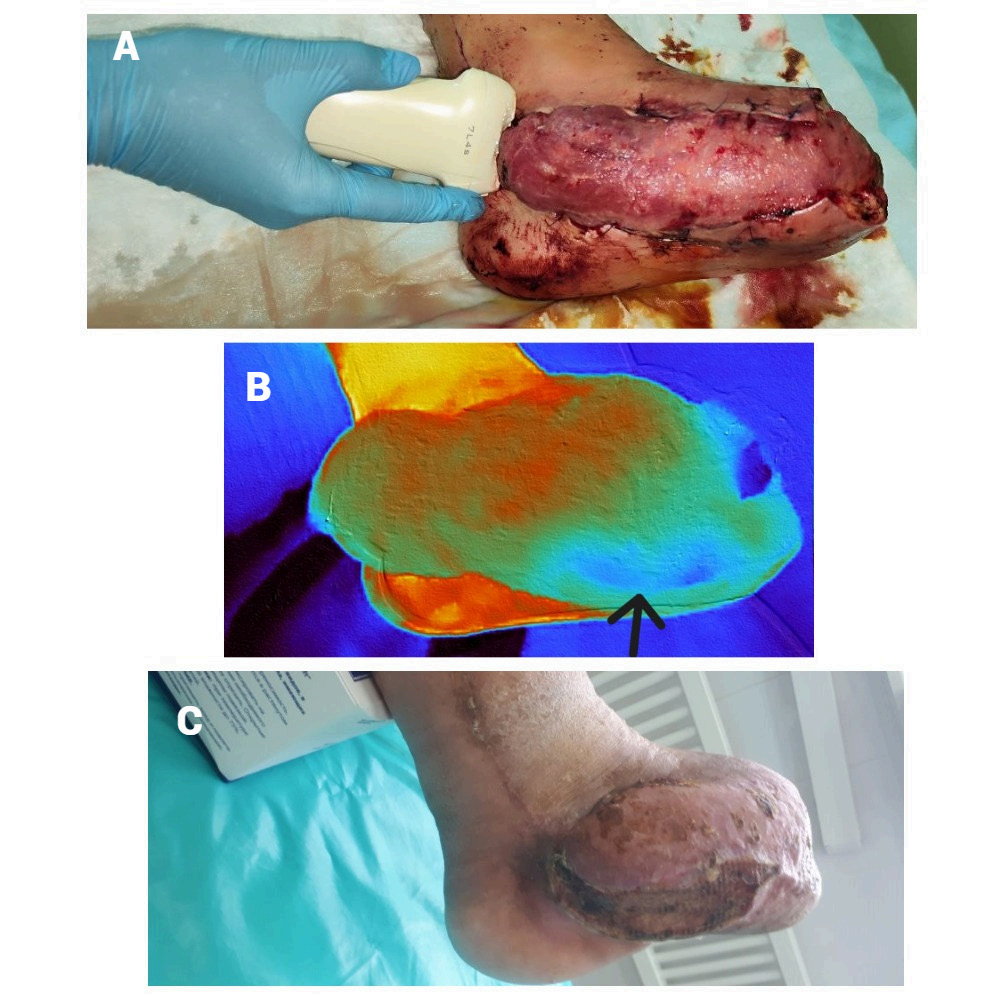
**(a)** Ultrasound monitoring of vascular pedicle patency; **(b)** thermography to assess flap viability; a hypoperfusion zone is visible, which subsequently developed partial necrosis; **(c)** result after 5 months.

The patient reported a gradual decrease in resting pain after surgery with complete relief after 6 weeks (Figure 2).

## CASE 2

A 53-year-old man with type 2 diabetes had wet necrosis of the forefoot, but did not complain of pain due to peripheral neuropathy. No changes of interest were noted in the analyses or the wound culture results. First, we performed femoral-PTA autovenous bypass, medial plantar artery balloon angioplasty, and necrectomy. Several weeks later, after the wound was cleared, the patient underwent free gracilis transfer to the forefoot. The flap artery was elongated by end-to-end anastomosis with a reversed autovein, which was transferred from the anterolateral surface of the leg through the interosseous membrane to the posterior tibial bypass and anastomosed with the latter end-to-side.

**Figure 3:**
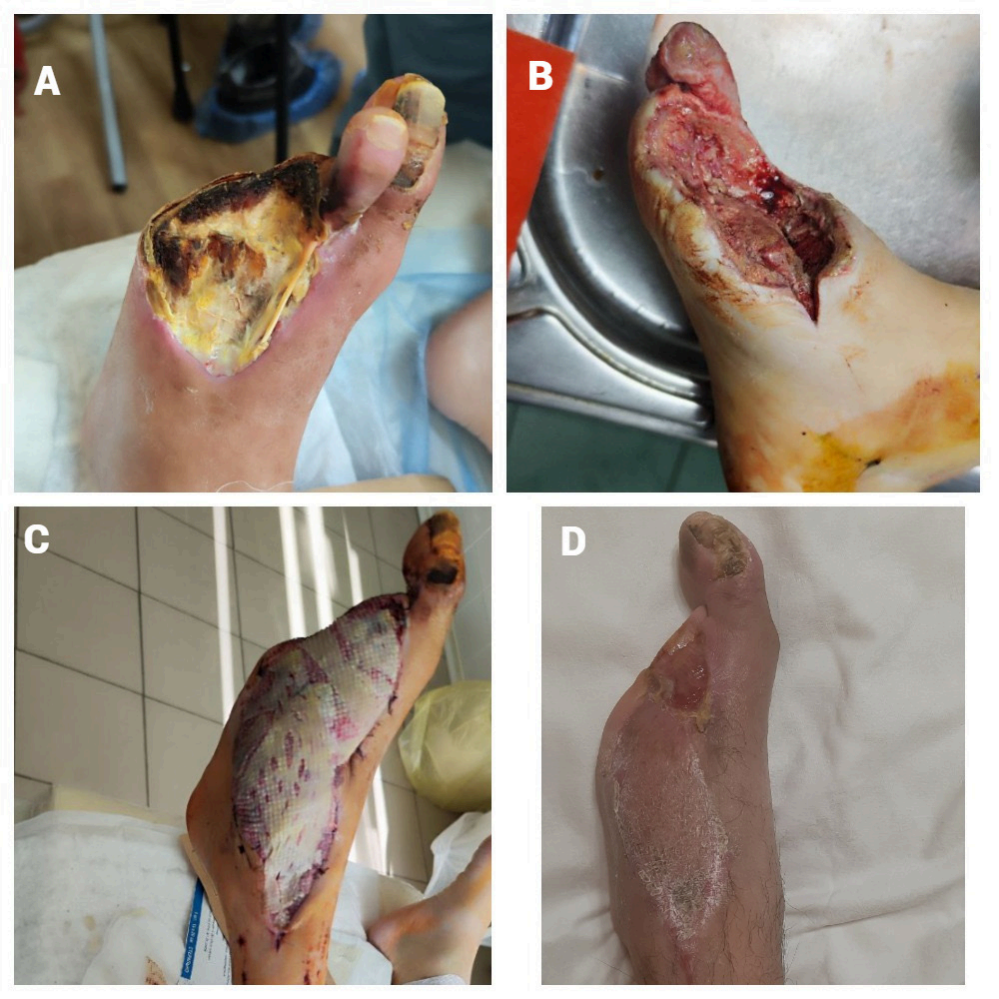
**(a)** Forefoot necrosis; **(b)** after surgical debridement; **(c)** wound after flap transplantation and autodermoplasty; **(d)** result 5 months later.

In the postoperative period, partial necrosis of the flap was noted, necessitating removal of the necrotic part of the muscle. After 1 month, the patient was actively walking and the distal part of the wound had become granular (Figure 3).

**Figure 4:**
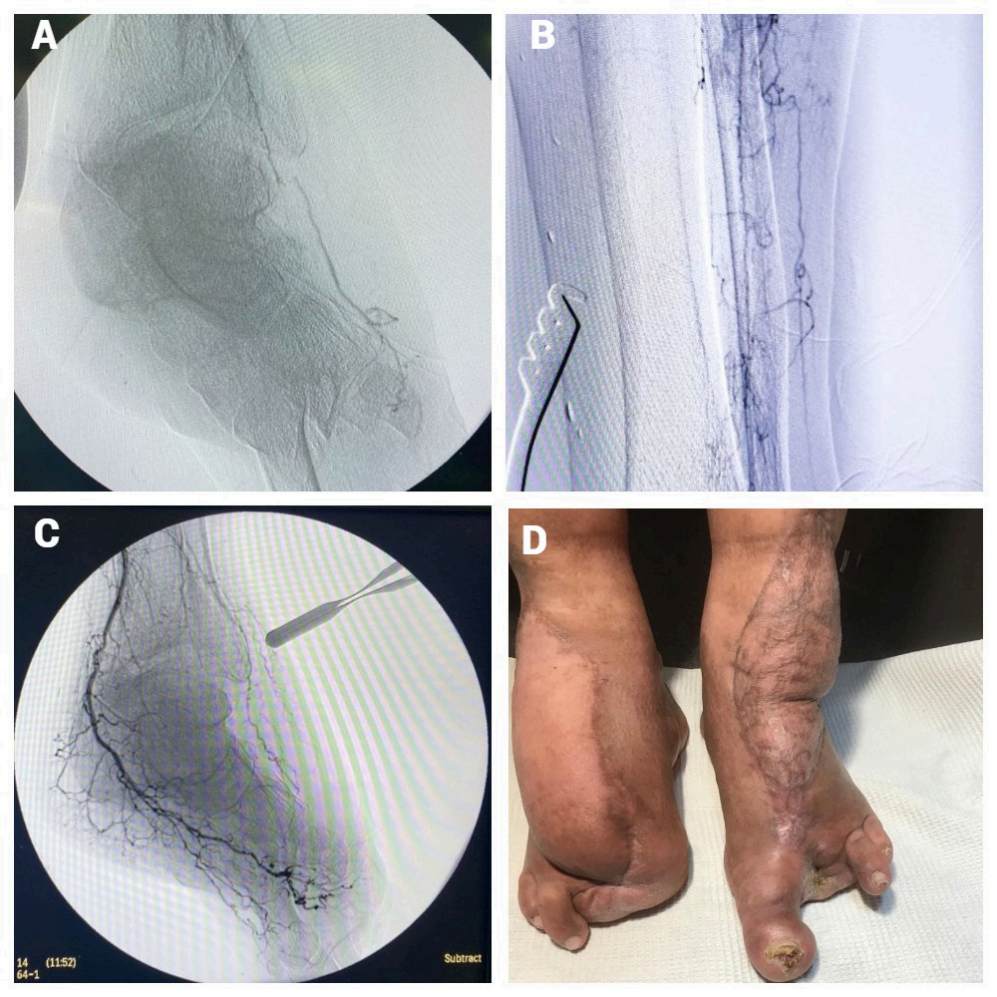
Case 2: **(a)** Angiography through the suboccluded autovenous insertion into the flap artery; **(b)** neovascularization around the autovenous insertion into the flap artery; **(c)** angiography via posterior tibial bypass, showing collateral filling of the flap; Case 3: **(d)** successful limb salvage by antero-lateral and gracilis flap transfer.

At the 6-month follow-up examination, stenosis of the posterior tibial bypass graft was detected at the site of anastomosis with the autovenous insertion (elongation) to the flap artery. The patient was hospitalized, and the stenosis zone was revised. The posterior tibial bypass graft was pulsating, unlike the insertion. The latter was transected and its lumen was found to be suboccluded. Intimal hyperplasia was detected at the anastomosis site. Angiography of the posterior tibial bypass was performed, followed by autovenous plastic reconstruction of the stenosis site. Angiography of the autovenous insertion was also performed, after which it was ligated. Finally, autodermoplasty of the granulating wound was carried out with a small split skin graft. Both angiographies showed filling of the flap vascular network (direct or collateral), indicating complete integration of the flap. Interestingly, significant neovascularization was observed around the autovenous insertion and the flap pedicle. There is an opinion that obliteration of the flap pedicle after its incorporation into the recipient network may be associated with excess blood flow to it (Figure 4).

## CASE 3

A 56-year-old male smoker, who also suffered from Buerger’s disease, was admitted with complaints of pain at rest in the left foot and acral necrosis. Three years prior to hospitalization, he had undergone a free anterolateralis fasciocutaneus flap transfer to the right foot, resulting in successful limb salvage. In this case, no interesting changes were found in the results of laboratory tests or wound cultures. CT analysis revealed absence of a left foot arterial bed with passable arteries in the lower third of the leg. An attempt at thrombectomy of the foot arteries was unsuccessful. We decided to transfer a free gracilis flap to the left foot, employing anastomoses of two flap veins and anterior tibial veins end-to-side, as well as flap arteries with anterior tibial artery end-to-end. Pain syndrome was resolved one month after surgery; seventeen months later, the wound had healed and the patient was walking well.

## DISCUSSION

Despite the fact that, according to international clinical guidelines, the optimal treatment for critical ischemia of the lower extremities is direct surgical or endovascular revascularization, in some patients, the absence of any arteries in the foot or compromise of the arterial bed in the foot, leading to high bypass resistance, makes such procedures impossible.^2^ In these cases, it is reasonable to attempt a shunt decompression or an indirect revascularization of the foot with a free flap, prior to amputation, which is associated with high mortality. The first report of successful salvage of the lower limb through a distal direct revascularization followed by plastic reconstruction of ischemic defects appeared in 1985 and the concept of a “nutrient flap” was developed in 1989.^3,4^ According to a meta-analysis, the limb preservation rate five years after successful flap transplantation is 71%.^5^ Some studies have demonstrated the insertion of the flap into the recipient zone, and even its functionality in cases of distant thrombosis of the bypass feeding the flap.^6-9^ In the second case described herein, even though the autovenous insertion to the flap artery became suboccluded, the flap had had time to integrate into the recipient bed, which was enough to maintain its viability; neoangiogenesis around the occluded flap pedicle is also interesting. Also, in this report we may add to the latter a gradual decrease in ischemic pain in patients suffering from Buerger’s disease in the absence of an arterial bed in the foot within 1 to 1.5 months after flap transplantation, which may also indirectly indicate a gradual integration of the flap into the recipient network.

Although various flaps are used in surgery, in our opinion, the gracilis muscle flap is advantageous because it can be harvested from the same limb, does not require additional anesthesia, and is relatively simple to obtain due to the anatomical constancy of the flap pedicle. Another considerable advantage is the absence of significant functional deficit in the donor area.

Among the contraindications for free flap transplantation, a pronounced infectious process in the recipient zone should be noted. It is also important to consider that in cases of significant damage to the distal arterial bed due to calcification or vasculitis, preoperative ultrasound examination of the flap pedicle should be conducted, and if compromised, a different type of free flap with a less affected vascular pedicle should be chosen. Although the flap pedicle usually has a stable anatomy, variations such as duplication or branching of the pedicle have been described in the literature, which may render further transplantation questionable or impossible.^10^ In the postoperative period, the flap often enlarges due to edema, but this should not cause much concern, as the swelling subsides after 12 months and flap remodeling occurs.^11^

In our opinion, free flap revascularization fully deserves to be considered a therapeutic option for patients with critical lower limb ischemia and so-called desert foot — the complete absence or compromise of arteries in the foot.

The photographs of patients’ wounds and limbs are published with their consent. This study was approved by the institutional ethics committee (Protocol No. 3/11 dated April 8, 2025). The manuscript complies with the Declaration of Helsinki and applicable local ethical guidelines.

## Data Availability

The data supporting this study are available upon request to the corresponding author, Kalitko I. I., due to ethical restrictions.
